# Use of Oxidative Stress Biomarkers in *Cyprinus carpio* L. for the Evaluation of Water Pollution in Ataturk Dam Lake (Adiyaman, Turkey)

**DOI:** 10.1007/s00128-013-1187-0

**Published:** 2014-01-01

**Authors:** Hasan Karadag, Özgür Fırat, Özge Fırat

**Affiliations:** 1 Department of Chemistry, Faculty of Science and Letters, Adiyaman University, 02040 Adiyaman, Turkey; 2 Department of Biology, Faculty of Science and Letters, Adiyaman University, 02040 Adiyaman, Turkey; 3 Vocational School of Kahta, Adiyaman University, 02400 Adiyaman, Turkey

**Keywords:** Ataturk Dam Lake, Wastewater, Fish, Blood, Oxidative stress biomarkers

## Abstract

Adiyaman city, which is located in the north of the Ataturk Dam Lake, has no wastewater purification facilities which results in municipal, agricultural, and industrial wastewater discharges directly entering the reservoir. To assess the pollution in the dam lake, we used several oxidative stress biomarkers in blood tissue of *Cyprinus carpio*. Fish samples were taken from Sitilce, polluted area by untreated wastewaters, and Samsat, relatively clean area, in the reservoir in August 2012. The activity of catalase and level of malondialdehyde increased while activity of superoxide dismutase and glutathione level decreased in fish from Sitilce site when compared to Samsat site. The findings of the present investigation suggest that the presence of certain prooxidative compounds that can lead to oxidative stress in the fish at the Sitilce site and oxidative stress biomarkers may be important in order to evaluate the effects of untreated wastewaters on living organisms in the dam lake.

The main source of freshwater pollution can be attributed to discharge of untreated waste, dumping of industrial effluent, and run-off from agricultural fields (Adeyemo 2005). The complex mixtures of pollutants in aquatic ecosystems may exert severe damage on the aquatic biota (Lopez-Lopez et al. 2011). Biomarkers, representing toxicant-induced changes in biological systems, can serve as links between an environmental contamination and its effects, providing therefore unique information on the ecosystem health (Maria et al. 2009). Many environmental pollutants or their metabolites are capable of inducing oxidative stress in aquatic organisms, including fish (Velkova-Jordanoska et al. 2008; Lopez-Lopez et al. 2011). Antioxidants such as catalase (CAT), superoxide dismutase (SOD), and glutathione (GSH) have been proposed as biomarkers of contaminant or seasonally mediated oxidative stress in a variety of marine and freshwater organisms and their induction reflects a response to pollutants (Borković et al. 2005).

The Ataturk Dam Lake (37°23′29″N, 38°34′38″E) was constructed on the Euphrates River in South-Eastern Anatolia for electric generation and irrigation purposes. The surface area and total water deposit of the reservoir are about 817 km^2^ and 48.7 billion m^3^, respectively. The dam lake is one of the largest artificial lakes of the Europe and Asian regarding surface area and hydropower production (Alp et al. 2010). It is an abundant source of food for local people and also provides opportunities for recreational fishing. Ten towns and 156 villages from three provinces are located around the dam lake. Recently, agricultural and industrial developments as well as increase in population have substantially increased the contamination of the reservoir (Karadede et al. 2004). According to our knowledge, there is no report on antioxidant responses of fishes to pollutants in the Ataturk Dam Lake. Therefore, the aim of this study was to explore whether the untreated wastewaters of Adiyaman city could induce oxidative stress and damage to aquatic organisms in the dam lake. Activities of SOD and CAT and levels of GSH and malondialdehyde (MDA) in the blood tissue of the *Cyprinus carpio* collected from Sitilce and Samsat sites were analyzed.

## Materials and Methods

**Fig. 1:**
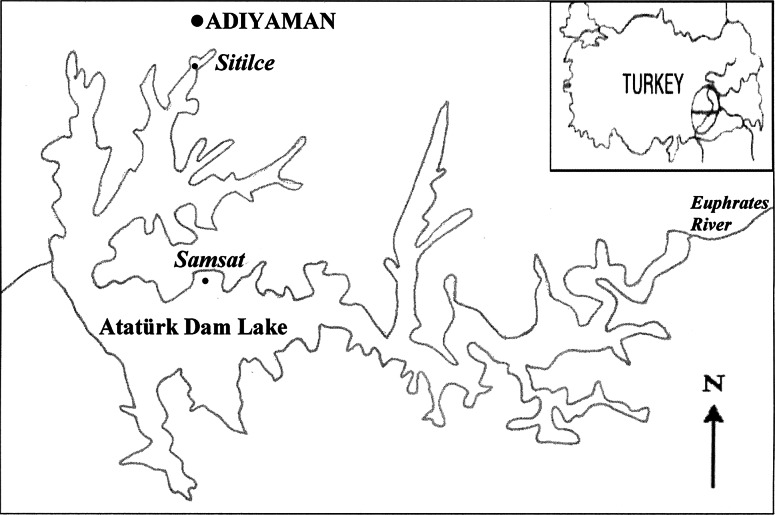
The studied sites (Sitilce and Samsat) in the Ataturk Dam Lake, Turkey

*Cyprinus carpio* is the most common fish in the Ataturk Dam Lake and is inevitably exposed to the pollutants in the reservior. Fish samples were collected from two study sites (Sitilce and Samsat) in the Northern parts of the dam lake in August 2012 (Fig. 1). The reservoir has an economical importance for fishery. Thus, contamination in the region is an important issue regarding the health of the aquatic animals and in turn, health of the human. Adiyaman city, an industrial and agricultural city located approximately 35 km north of the dam lake, has no wastewater purification facilities which results in municipal, agricultural, and industrial effluents directly entering the dam lake. The Sitilce site is polluted by the industrial and municipal wastewater discharges whereas the Samsat site is a relatively clean region because it is not affected directly or indirectly by anthropogenic wastes.

Some physico-chemical parameters of water were measured at each sampling site. Water temperature, dissolved oxygen concentrations, and pH were recorded in the field using portable meters. The other water quality parameters such as ammonia, nitrite, nitrate, sulphate, phosphate content were measured according to Standard Methods (APHA 1998).

Ten specimens from each sampling site were caught using fishing nets. All fishes were collected from a single fisherman in order to assure regularity in fishing methods. The mean values and ± standard errors of the size and weight of the fish were recorded as follows: 40.90 ± 0.77 cm and 1,045 ± 44 g for Samsat site, 40.50 ± 0.70 cm and 994 ± 52 g for Sitilce site, respectively. Blood samples were taken from the caudal vein of each fish into tubes containing EDTA as anticlotting agent and were immediately transported on ice into the laboratory. Some of the whole blood was used immediately to determine CAT and SOD activities and GSH level. Remaining whole blood was centrifuged at 5,000 rpm for 10 min at 4°C and plasma samples were stored at −80°C until MDA analysis. All chemical used in this study was obtained from Sigma or Merck (Germany).

Glutathione was measured following the method of Beutler (1975). CAT and SOD activities were measured according to the methods of Lartillot et al. (1988) and Sun et al. (1988), respectively. MDA level was assayed by the method of Dubovskiy et al. (2008). The blood protein contents were determined using the method of Lowry et al. (1951). Data are presented as mean ± standard error. For the statistical analysis, it was used the independent sample *t* test for comparing the study sites. Differences were considered significant if *p* < 0.05.

## Results and Discussion

Physico-chemical parameters of water samples from two sites are presented in Table 1. The values of pH, ammonia, nitrite, nitrate, sulphate, and phosphate were higher whereas the value of dissolved oxygen level was lower for the water taken from Sitilce polluted by wastewaters compared to those collected from the Samsat site. The activities of antioxidant enzymes and levels of GSH and MDA in blood of *C. carpio* caught from the Ataturk Dam Lake are shown in Table 2. When compared with fish taken from the Samsat site, it was observed that the CAT activity and MDA level were higher, whereas the SOD activity and GSH level were lower for the fish taken from the Sitilce site. The increases of CAT activity and MDA level were 82.6 % and 171.2 %, respectively, while the decreases of SOD activity and GSH level were 49.4 % and 62.8 %. The observed results indicate that the Sitilce site should be considered as an area polluted by the wastewater discharges from Adiyaman city. Previous studies analyzed the concentrations of Cd, Pb, Cr, Co, Ni, Cu, Zn, Fe, and Mn in water and the gill, liver, and muscle tissues of fish species, *C. carpio* and *Capoeta trutta*, taken from the Ataturk Dam Lake in August 2009 and found that due to municipal and industrial effluents all metal levels in water and fish samples were significantly higher at the Sitilce site than at the Samsat site (Fırat et al. 2010). These results suggest that the presence of heavy metals, that have been reported to cause oxidative stress (Dimitrova et al. 1994; Fırat et al. 2010), may be associated with the results found in the present study. In agreement with the present results, Yildirim et al. (2011) reported increased CAT activity and MDA level and decreased GSH level in tissues of *C. trutta* caught from contaminated site compared to those collected from the uncontaminated site in Munzur River (Turkey). The researchers concluded that alteration in the antioxidant enzymes, glutathione system and induction of lipid peroxidation (LPO) reflects the presence of pollution, which may cause oxidative stress in the *C. trutta* from Munzur River.

**Table 1:** Values of some physical and chemical parameters of water from Atatürk Dam Lake

Site	pH	Temperature (°C)	Dissolved oxygen (mg/L)	Ammonia (mg/L)	Nitrite (mg/L)	Nitrate (mg/L)	Sulfate (mg/L)	Phosphate (mg/L)
Samsat	8.05	26.48	7.11	0.02	0.02	0.07	21.00	3.00
Sitilce	8.32	26.54	3.87	0.14	0.12	0.67	39.00	9.00

**Table 2:** Oxidative stress parameters in blood of *C. carpio* collected from two sites in Atatürk Dam Lake

Oxidative stress parameters	Samsat site	Sitilce site
CAT (U/mg protein)	12.33 ± 0.67	22.52 ± 0.58*
SOD (U/mg protein)	0.77 ± 0.02	0.39 ± 0.01*
GSH (μmol/g protein)	3.98 ± 0.14	1.48 ± 0.10*
MDA (nmol/g protein)	14.66 ± 0.77	39.76 ± 1.37*

Values are expressed as mean ± standard error (n = 10)

* *p* < 0.05; (independent sample *t* test) statistical differences between sites

The release of pollutants into the aquatic environment is known to cause detrimental effects to the environment and to the living organisms, giving a significant interest to the study of oxidative stress responses in aquatic organisms induced by toxicants (Soares et al. 2008). Many pollutants can result in some degree of oxidative damage by generating free radicals and/or altering antioxidant enzyme systems which reactive oxygen species (ROS) (Huang et al. 2007). Antioxidant defence enzymes have been proposed as biomarkers of contaminant or seasonally mediated oxidative stress in a variety of marine and freshwater organisms and their induction reflects a specific response to pollutants (Borković et al. 2005). Of these enzymes, SOD which catalyses the conversion of the superoxide anion radical to molecular oxygen and hydrogen peroxide (H_2_O_2_) has been called the cell’s first defense line against ROS and could protect against superoxide-induced oxidative damage (Fridovich 1989). CAT is a well-known antioxidative enzyme and has been implicated in protection against H_2_O_2_. In the present study the significant decrease/increase in activities of the SOD and CAT enzymes in fish from the Sitilce site may be related to pollutants that increase ROS production resulting in oxidative stress. Usually a simultaneous induction response in the activities of SOD and CAT is observed when exposed to pollutants (Dimitrova et al. 1994). However, in our study no such relationship was shown.

Catalase activity increase suggests the presence of higher peroxide concentrations (Carvalho et al. 2012). The biological importance of CAT is more evident from various studies due to the fact that H_2_O_2_ is the main cellular precursor of the hydroxyl radical which is a highly reactive and toxic form of ROS (Vlahogianni et al. 2007). The removal of H_2_O_2_ is an important strategy of aquatic organisms against oxidative stress. Therefore the observed increase in CAT activity may indicate an important role to protect cells against H_2_O_2_ production. Since superoxide radical is a precursor to several other highly reactive species, control of this free radical concentration by SOD constitutes an important protective mechanism (Fridovich 1989). SOD catalytically scavenges superoxide radical which appears to be an important agent of toxicity of oxygen and this provides a defense against this aspect of oxygen toxicity (Kadar et al. 2005). In this work a decline in SOD activity may show a reduced ability to protect cells against superoxide radicals. Also, Ozmen et al. (2004) suggested the depression in SOD activity may result in cellular injury by superoxide radical. In order to verify the effects of DDT and MeHg on fish *Hoplias malabaricus* hepatocytes, intracellular concentrations of ROS, and SOD and CAT activities were measured and it was observed that hydrogen peroxide and superoxide anion levels increased (Neto et al. 2008). The researches reported an increase in CAT activity and a decline in SOD activity due to elevated ROS levels. The work by Yin et al. (2007) showed that phenanthrene could be accumulated in liver of fish (*Carassius auratus*) and induce ROS production, leading to oxidative stress and the changes in the activities of the antioxidant enzymes also confirmed it. Similar to the results of the present study, Lopez-Lopez et al. (2011) found that *Goodea atripinnis* exposed to water samples of Lake Yuriria polluted by domestic sewage, industrial effluents, and municipal wastewaters displayed decreased SOD activity and increased CAT activity. They concluded that the decline in SOD activity might reflect damage to the SOD protein due to ROS overproduction and the elevation in CAT activity showed a relevant role to face oxidative stress in fish. Also, Dimitrova et al. (1994) suggested that the excess production of superoxide radicals by themselves or after their transformation to H_2_O_2_ causes the oxidation of the cysteine in SOD that deactivates it. The elevated CAT activity in *C. carpio* from Sitilce site indicates that CAT activity could be induced to resist the pollutants toxicity. CAT is so efficient that it cannot be saturated by H_2_O_2_ at any concentration (Mates and Sanchez-Jimenez 1999), a characteristic that allows it to play an important role in the acquisition of tolerance to oxidative stress (Hunt et al. 1998). The work by Carvalho et al. (2012) showed a decline in SOD activities in liver and white muscle and an elevation in CAT activity in gill of *Oreochromis niloticus* collected from polluted site in Monjolinho River (São Carlos, SP, Brazil). The study by Velkova-Jordanoska et al. (2008), assessing the oxidative effects of anthropogenic pollutants on fish (*Barbus m. petenyi* Heck.) living in Lake Ohrid (Macedonia), indicated that decreased SOD activity and increased CAT activity were observed in blood of fish due to toxic impact of the pollutant in the aquatic environment.

Glutathione is a tripeptide nonenzymatic antioxidant with a single cysteine residue and constitutes an important pathway of the antioxidant and detoxification defenses (Vlahogianni et al. 2007). However, under severe oxidative stress GSH levels are suppressed due to the loss of compensatory responses and oxidative conversion of GSH to its oxidised form (Chen and Lin 1977). A decline in the GSH level in the blood of *C. carpio* taken from the Sitilce site was shown in our study, which may be attributed to the influence of various organic and inorganic components in this site. Our results are similar to those of Mather-Mihaich and DiGiulio (1986), who found a decrease in GSH level in channel catfish exposed to bleached kraft mill effluent. Also, the GSH contents in *O. niloticus* liver diminished in the first week of exposure to the effluents derived from swine industry (Lima et al. 2006). GSH depletion seems to reflect an aggravation status due to reduced cell protection ability (Yin et al. 2011). Zhang et al. (2004) suggested that a severe oxidative stress may suppress GSH levels due to the impairment of adaptive mechanisms. Therefore decreased GSH level in the fish from Sitilce site might indicated that the abilities to protect against toxicants were reduced. The work by Yildirim et al. (2011) showed a decline in GSH level in tissues of *C. trutta* caught from contaminated site in Munzur River. They concluded that decreased GSH content during exposure to pollution may be due to an increased utilization of GSH, which can be converted into oxidized glutathione, and inefficient GSH regeneration.

Malondialdehyde is one of the LPO products deriving from oxidative attack on cell membrane phospholipids and circulating lipids, and its level directly reflects the degree of oxidative damage induced by contaminants (Banerjee et al. 1999). The measurement of MDA content (an index of LPO) provides a relative measure of the potential for pollutants to cause oxidative injury (Vlahogianni et al. 2007). The elevated MDA level was considered as a result of oxidative stress from xenobiotics in Sitilce site. Similar results were obtained for specimen of freshwater fish (Cyprinidae), as demonstrated by Gül et al. (2004). The researchers reported that the MDA level was increased in liver of fish collected from polluted areas in dam Lake of Seyhan, Turkey. SOD and GSH are among the most important antioxidants protecting from oxidative attacks by active oxygen species such as LPO, because they act as a reducing agent and free-radical trapper. Therefore the decreased SOD activity and GSH level in the blood of fish taken from the Sitilce site may demonstrate the inefficiency of this tissue in neutralizing the impact of ROS, resulting in increased LPO. Radwan et al. (2010) suggested that significantly elevated levels of LPO in the digestive gland of *Theba pisana* snails in response to toxicants indicate that some cell damage might have occurred.

To the best of our knowledge, this is the first report of enzymatic/non-enzymatic defence system as well as LPO in fish living in the Ataturk Dam Lake. Our study may suggest that untreated wastewaters from Adiyaman city caused a significant oxidative stress by disregulation in the antioxidant system and *C. carpio* in Sitilce site were undergoing this stress. The oxidative changes observed in the fish may indicate a potential health hazard of the wastewaters to aquatic organisms in the dam lake. Thus, it is possible that a potentially prospective pollution in this reservoir may affect not only these organisms but also people living near the dam lake through several avenues including water and food consumption. Therefore pollution prevention measures must be taken without delay to prevent harmful effects of wastewaters on aquatic biota of this reservoir. In conclusion, findings of the present investigation suggest that the presence of certain prooxidative compounds that can lead to oxidative stress in the fish at the Sitilce site and oxidative stress biomarkers may be important in order to evaluate the effects of untreated wastewaters on living organisms in the dam lake.

## References

[CR1] Adeyemo OK (2005). Haematological and histopathological effects of cassava mill effluent in *Clarias gariepinus*. Afr J Biomed Res.

[CR2] Alp MT, Kocer MAT, Sen B, Ozbay O (2010). Water quality of surface waters in lower Euphrates basin (Southeastern Anatolia, Turkey). J Anim Vet Adv.

[CR3] APHA (1998). Standard methods for the examination of water and wastewater.

[CR4] Banerjee BD, Seth V, Bhattacharya A (1999). Biochemical effects of some pesticides on lipid peroxidation and free-radical scavengers. Toxicol LetT.

[CR5] Beutler E (1975). Red cell metabolism: a manual of biochemical methods.

[CR6] Borković SS, Šaponjić JS, Pavlović SZ, Blagojević DP, Milošević SM (2005). The activity of antioxidant defence enzymes in the mussel *Mytilus galloprovincialis* from the Adriatic Sea. Comp Biochem Physiol.

[CR7] Carvalho CS, Bernusso VA, Araújo HSS, Espíndola ELG, Fernandes MN (2012). Biomarker responses as indication of contaminant effects in *Oreochromis niloticus*. Chemosphere.

[CR8] Chen LH, Lin SM (1977). Modulation of acetaminophen-induced alteration of antioxidant defense enzymes by antioxidants or glutathione precursors in cultured hepatocytes. Biochem Arch.

[CR9] Dimitrova MST, Tsnova V, Velcheva V (1994). Combined effect of zinc and lead on the hepatic superoxide dismutase-catalase system in carp (*Cyprinus carpio*). Comp Biochem Physiol.

[CR10] Dubovskiy IM, Martemyanov VV, Vorontsova YL, Rantala MJ, Gryzanova EV, Glupov VV (2008). Effect of bacterial infection on antioxidant activity and lipid peroxidation in the midgut of *Galleria mellonella* L. larvae (Lepidoptera, Pyralidae). Comp Biochem Physiol.

[CR11] Fırat Ö, Kargin F, Korkmaz S (2010) Determining of heavy metal levels *Cyprinus carpio* and *Capoeta trutta,* economical important species, from Atatürk Dam Lake. Final report of Scientific Research Projects of Adiyaman University, Project Number FEFBAP2008/0004, Adiyaman, pp 1–16

[CR12] Fridovich I (1989). Superoxide dismutases. An adaptation to a paramagnetic gas. J Biol Chem.

[CR13] Gül Ş, Kurutaş EB, Yıldız E, Şahan A, Doran F (2004). Pollution correlated modifications of liver antioxidant systems and histopathology of fish (Cyprinidae) living in Seyhan Dam Lake, Turkey. Environ Int.

[CR14] Huang DJ, Zhang YM, Song G, Long J, Liu JH, Ji WH (2007). Contaminants-induced oxidative damage on the carp *Cyprinus carpio* collected from the upper Yellow River, China. Environ Monit Assess.

[CR15] Hunt C, Sim JE, Sullivan SJ, Featherstone T, Golden W (1998). Genomic instability and catalase gene amplification induced by chronic exposure to oxidative stress. Cancer Res.

[CR16] Kadar E, Costa V, Santos RS (2005). Distribution of micro-essential (Fe, Cu, Zn) and toxic (Hg) metals in tissues of two nutritionally distinct hydrothermal shrimps. Sci Total Environ.

[CR17] Karadede H, Oymak SA, Ünlü E (2004). Heavy metals in mullet, *Liza abu*, and catfish, *Silurus triostegus*, from the Atatürk Dam Lake (Euphrates), Turkey. Environ Int.

[CR18] Lartillot S, Kadziora P, Athios A (1988). Purification and characterization of new fungal catalase. Prep Biochem.

[CR19] Lima PL, Benassi JC, Pedrosa RC (2006). Time course variations of DNA damage and biomarkers of oxidative stress in tilapia (*Orechromis niloticus*) exposed to effluents from a swine industry. Arch Environ Contam Toxicol.

[CR20] Lopez-Lopez E, Sedeno-Diaz JE, Soto C, Favari L (2011). Responses of antioxidant enzymes, lipid peroxidation, and Na^+^/K^+^-ATPase in liver of the fish *Goodea**atripinnis* exposed to Lake Yuriria water. Fish Physiol Biochem.

[CR21] Lowry OH, Rosebrough NJ, Farra NJ, Randall RJ (1951). Protein measurements with the Folin phenol reagent. J Biol Chem.

[CR22] Maria VL, Ahmad I, Oliveira M, Serafimb A, Bebianno MJ, Pacheco M, Santos MA (2009). Wild juvenile *Dicentrarchus labrax* L. liver antioxidant and damage responses at Aveiro Lagoon, Portugal. Ecotoxicol Environ Safe.

[CR23] Mates JM, Sanchez-Jimenez F (1999). Antioxidant enzymes and their implications in pathologyc process. Front Biosci.

[CR24] Mather-Mihaich E, DiGiulio RT (1986). Antioxidant enzyme activities and malondialdehyde, glutathione and methemoglobin concentrations in channel catfish exposed to DEF and n-butyl mercaptan. Comp Biochem Physiol.

[CR25] Neto FF, Zanata SM, Silva de Assis HC, Nakao LS, Randi MAF, Oliveira Ribeiro CA (2008). Toxic effects of DDT and methyl mercury on the hepatocytes from *Hoplias malabaricus*. Toxicol In Vitro.

[CR26] Ozmen I, Bayır A, Cengiz M, Sirkecioglu AN, Atamanalp M (2004). Effects of water reuse system on antioxidant enzymes of rainbow trout (*Oncorhynchus mykiss* W., 1792). Vet Med.

[CR27] Radwan MA, El-Gendy KS, Gad AF (2010). Oxidative stress biomarkers in the digestive gland of *Theba pisana* exposed to heavy metals. Arch Environ Contam Toxicol.

[CR28] Soares SS, Martins H, Gutierrez-Merino C, Aureliano M (2008). Vanadium and cadmium in vivo effects in teleost cardiac muscle: metal accumulation and oxidative stress markers. Comp Biochem Physiol.

[CR29] Sun Y, Oberley LW, Li Y (1988). A simple method for clinical assay of superoxide dismutase. Clin Chem.

[CR30] Velkova-Jordanoska L, Kostoski G, Jordanoska B (2008). Antioxidative enzymes in fish as biochemical indicators of aquatic pollution. Bulg J Agric Sci.

[CR31] Vlahogianni T, Dassenakis M, Scoullos MJ, Valavanidis A (2007). Integrated use of biomarkers (superoxide dismutase, catalase and lipid peroxidation) in mussels *Mytilus galloprovincialis* for assessing heavy metals’ pollution in coastal areas from the Saronikos Gulf of Greece. Mar Pol Bul.

[CR32] Yildirim NC, Benzer F, Danabas D (2011). Evaluation of environmental pollution at Munzur River of Tunceli applying oxidative stress biomarkers in *Capoeta trutta* (Heckel, 1843. J Anim Plant Sci.

[CR33] Yin Y, Jia H, Sun Y, Yu H, Wang X, Wu J, Xue Y (2007). Bioaccumulation and ROS generation in liver of *Carassius auratus*, exposed to phenanthrene. Comp Biochem Physiol.

[CR34] Yin F, Peng S, Sun P, Shi Z (2011). Effects of low salinity on antioxidant enzymes activities in kidney and muscle of juvenile silver pomfret Pampus argenteus. Acta Ecol Sin.

[CR35] Zhang J, Shen H, Wang X, Wu J, Xue Y (2004). Effects of chronic exposure of 2,4-dichlorophenol on the antioxidant system in liver of freshwater fish Carassius auratus. Chemosphere.

